# Remote assessment of surgical site infection (SSI) using patient-taken wound images: Development and evaluation of a method for research and routine practice

**DOI:** 10.1016/j.jtv.2023.01.001

**Published:** 2023-02

**Authors:** Rhiannon C. Macefield, Jane M. Blazeby, Barnaby C. Reeves, Anni King, Jonathan Rees, Anne Pullyblank, Kerry Avery

**Affiliations:** a National Institute for Health and Care Research (NIHR) Bristol Biomedical Research Centre (BRC), Population Health Sciences: Bristol Medical School, University of Bristol, UK; b Bristol Trials Centre, Bristol Medical School, University of Bristol, UK; c Hepatobiliary Surgery, University Hospitals Bristol NHS Foundation Trust, UK; d Bristol Medical School, University of Bristol, UK; e Department of Surgery, North Bristol NHS Trust, UK

**Keywords:** Surgical site infection, Wound assessment, Wound images, Remote follow-up, Telemedicine

## Abstract

**Background/aim:**

Clinical assessment of wounds for surgical site infection (SSI) after hospital discharge is challenging and resource intensive. Remote assessment using digital images may be feasible and expedite SSI diagnosis. Acceptable and accurate methods for this process are needed. This study developed and evaluated the feasibility, acceptability and usability of a method for patients to capture standardised wound images for remote wound assessment to detect SSI.

**Materials and methods:**

The work was conducted in two phases. Phase I involved: i) a review of literature to identify key components of photography relevant to taking wound images, ii) development of wound photography instructions for patients and a secure process for transmission of images using electronic survey software and iii) pre-testing of the photography instructions and processing method with a sample of 16 patients using cognitive interviews and observations. Phase II involved a prospective cohort study of 89 patients to evaluate the feasibility, acceptability and usability of the remote method following discharge from hospital after surgery. Quality of the images was assessed by three independent clinical reviewers.

**Results:**

Some 21 key components for photographing wounds were identified from 11 documents. Of these, 16 were relevant to include in instructions for patients to photograph their wounds. Pre-testing and subsequent iterations improved understanding and ease of use of the instructions and the process for transmitting images. Fifty-two of 89 (58.4%) patients testing the method remotely took an image of their wound(s) and 46/52 (88.5%) successfully transmitted images. When it was possible to ascertain a reason for not taking/transmitting images, this was primarily health problems (n = 7) or lack of time/poor engagement with the study (n = 4) rather than problems relating to technology/competency (n = 2) or practical issues relating to the wound itself (n = 2). Eighty-seven (85.3%) of the 102 images received were evaluated to be of high quality and sufficient to remotely assess SSI by at least two independent reviewers.

**Conclusion:**

A simple, standardised and acceptable method for patients to take and transmit wound images suitable for remote assessment of SSI has been developed and tested and is now available for use in routine clinical care and research.

## Highlights

• Remote wound assessment using images is a feasible option for research studies and routine clinical care.
• A method for patients to take and transmit wound images suitable for remote SSI assessment has been developed and tested.
• Photography instructions for patients were demonstrably effective in producing high quality images to assess the wound.
• Photography instructions and process to transmit images are reproducible and ready for use in research and routine follow-up.

## 1 Introduction

Surgical site infection (SSI) is one of the most common adverse events after surgery [1,2] and can result in substantial patient morbidity and costs to health services. Reduction of SSI rates is a high priority worldwide with many countries investing in SSI surveillance systems and studies of preventive interventions [3,4]. However, with increasingly shorter stays in hospital after surgery, assessment of SSI is challenging because SSI presents most often after a patient is discharged from hospital [1,5,6].

Traditionally, wound assessment and SSI diagnosis have been conducted in-person by a trained healthcare professional, typically within 30-days after surgery. This is resource intensive and expensive for healthcare providers. Attending face-to-face appointments may also be logistically and practically difficult for patients if they need to travel or take time off work, or if they have mobility or health issues. Remote wound assessment using digital technology is a feasible option for research studies and routine clinical care to overcome these issues. Remote assessment has benefits where there is a need to minimise face-to-face appointments to reduce the burden on staff and healthcare resources or reduce transmission of viruses, as underlined by the recent COVID-19 pandemic. Huge advantages of remote assessment particularly apply to low- and middle-income countries (LMICs) where travel from rural settings to hospital for follow-up after surgery can be extremely challenging with logistic and financial constraints [7]. Remote assessment provides an opportunity for the healthcare team to check for infections and prevent a minor problem (that may not be sufficient for a patient to seek to travel back to hospital) developing into a major one, and avoid unnecessary suffering in the community. Regardless of the setting (i.e. LMIC or high-income countries), the opportunity for remote assessment may reduce patients’ anxiety if they are concerned about how the wound is healing and want to seek advice without having to schedule a face-to-face appointment.

The UK National Health Service (NHS) is planning to fund and introduce digital health technologies to optimise patient follow-up and reduce patient and resource burden [8,9]. Research studies are implementing remote follow-up (using telephone and/or video call assessment) in global SSI trials, and systematic reviews have been conducted to evaluate its effectiveness [7,10]. The use of digital wound images, however, taken by patients and/or carers after hospital discharge is a potential method for remote SSI diagnosis and/or surgical follow-up, with emerging evidence of the benefits and acceptability to patients [[11], [12], [13], [14]]. Images have the advantage of providing a permanent record that can be used to monitor wound healing, with evidence of increased specificity and improved confidence in SSI diagnoses compared to scenarios using data from hospital records alone [15,16]. Images of wounds may supplement patient-reported signs and symptoms to aid remote assessment [[17], [18], [19], [20], [21], [22]].

The increasing ownership of, or access to, personal mobile devices with built-in cameras provides an opportunity to advance the way in which wounds can be assessed remotely after a patient has been discharged [21,22]. Such devices provide the facility for patients to take and transmit digital wound images to healthcare providers or researchers after leaving hospital. A small number of studies have been reported to examine the potential of patient-submitted wound images for post-operative follow-up, many of these involving a bespoke mobile application [[11], [12], [13], [16], [23], [24], [25]]. A literature review of the use of patient-generated health data for post-operative care and remote SSI surveillance, conducted as part of a Health Technology Assessment in the US (Assessing Surgical Site Infection Surveillance Technologies; ASSIST study) found patient-submitted images aided in identification of post-operative complications and reducing readmissions [26,27]. The ASSIST project included a technical and market review of 11 identified patient-centred apps, with eight apps including transmission of wound images [26]. Qualitative work to explore patients’ views about the acceptability of an app, including wound photography, for post-operative SSI monitoring found the idea highly acceptable [14]. The development of apps, however, comes with a burden of cost and resource use implications. 10.13039/100014337Furthermore, patients may not always have the appropriate mobile devices to support the app [28]. In the UK, a recent randomised controlled trial of 492 patients undergoing emergency surgery demonstrated that a smartphone tool for patients to self-report SSI symptoms including transmission of images of wounds increased the likelihood of SSI diagnosis within the early period (7 days) following surgery compared to routine follow-up (i.e., no smart-phone tool intervention). Incorporation of images offered a significant improvement in specificity of diagnostic accuracy of SSI diagnosis (from 84.4% to 93.6%) compared to patient-reported symptoms alone [23]. Lacking from these reports, however, is information on how patients were instructed to take the wound images, with only one study identified that provided written instructions for patients [16]. It is unknown whether photography instructions for patients were adequate to enable patients to obtain images that were suitable and of sufficient quality to be clinically useable to assess the wound for SSI.

Guidelines and standard operating procedures (SOPs) on how to take a clear, standardised wound images of a wound exist for trained professionals, for example, medical photographers and clinical staff [[29], [30], [31]]. However, these are primarily for use by healthcare professionals and not by patients. Furthermore, they have been developed for use with high-quality digital cameras in clinical rather than remote settings. Robust methodological work is needed to develop and test photography instructions for patients. Furthermore, work to explore whether it is feasible and acceptable to patients to take and transmit a wound image using their own mobile devices in a remote setting, without the use of an app, and whether images are of sufficient quality for wound assessment is required. The aim of this study was to i) develop instructions for patients or family members/caregivers to take a clear, standardised image of the surgical wound after hospital discharge and a process for transmitting them, and ii) evaluate the acceptability and usability of the method and quality of the images for remote SSI assessment.

## 2 Methods

This study was conducted in two phases, drawing on methodological approaches for developing and validating patient-reported outcome measures and evaluating human-system interaction [[32], [33], [34]]. Phase I involved a literature review to identify the content for wound photography instructions for patients, followed by cognitive interviews and observations to pre-test and refine the instructions and the process to transmit images. Phase II was a prospective cohort study to evaluate the method remotely and assess image quality.

Patient and public input towards the study concept, design and development of study materials was sought. Ethics approval was granted by the UK Health Departments Research Ethics Service NHS REC West Midlands - Coventry and Warwickshire Research Ethics Committee (18/WM/0096). Written informed consent was taken from all patients.

### 2.1 Phase I - development and pre-testing

#### 2.1.1 Literature review to identify key components of wound photography

Existing wound photography literature was examined to identify key components for producing a clear, standardised image of the wound. Literature was identified via a scoping search for published documents on the use of digital images in wound care. Further literature (published and unpublished documents) from studies of wound care interventions that used images for outcome assessment was identified through networks and colleagues known to the study team. Documents were obtained from online sources or by contacting relevant study teams.

Data on how to take a standardised wound image were extracted as verbatim text and categorised based on the component of photography that they related to (for example, lighting or framing of the image). Categories were modified and adapted in an iterative, inductive manner throughout the categorisation process [35]. Data extraction and categorisation was performed by one reviewer, in discussion with the wider study team.

#### 2.1.2 Drafting of photography instructions for patients and process for transmitting images

Identified components of photography were examined for their relevance to include in photography instructions for patients, with consideration of application in a non-clinical setting and the use of a camera on a mobile device. Relevant components were formulated into preliminary step-by-step instructions for patients to take a standardised image of their wound using their own smartphone or mobile device. Accompanying general information was also drafted, including information about the intended reason for taking a photograph of the wound, devices that could be used, and that help from others to take the image could be sought. All instructions were written in plain language. Members of the study team (AK and JR; a medical photographer and clinical consultant) were consulted to ensure that the instructions for patients met clinical and practical requirements for photographing wounds.

Existing secure online survey software with a facility to upload images was utilised for the process of transmitting images (Research Electronic Data Capture (REDCap) software) [36]. Features included a secure web application with suitable interfaces for collecting data using a range of mobile devices, including smart phones and tablet computers; of particular importance for the purpose of this study where a range of patients’ own devices may be used. The software included the facility to send an automatically generated personalised hyperlink via email for individuals to directly enter and upload data in the form of an online survey. A preliminary version of the survey, including step-by-step written instructions on how to upload and transmit images stored on the mobile device, was developed by the study team in conjunction with an expert REDCap data manager.

#### 2.1.3 Pre-testing the photography and processing method

Pre-testing of the wound photography instructions and process to transmit images was undertaken using in-person one-to-one cognitive interviews and observation [37,38]. Participants were patients who had recently undergone a broad range of general abdominal or vascular surgeries, with wounds primarily on the abdomen, groin area or lower limbs. Patients were recruited from pre- and post-surgical clinics and wards by research nurses and surgeons within two UK National Health Service (NHS) hospital trusts. Patients were purposively sampled to ensure diversity across a range of demographic and surgical characteristics including age and size and location of the wound. Recruitment was undertaken between June and November 2018. Written informed consent was taken by the study researcher at the time of the pre-testing session.

A pre-testing session with the study researcher was arranged for each participant after they had been discharged from hospital, to take place in their preferred setting (i.e., their own home or other suggested location). During the session, participants were provided with a paper-copy of the photography instructions and were requested to follow them to take an image of their surgical wound(s). A hyperlink to access the online survey was then sent to the participant by email during the same session, and participants were requested to open this and follow the process for uploading their images(s). The study researcher observed throughout. Cognitive interviews with participants were conducted and participants were asked to ‘think aloud’ and verbalise any thoughts throughout the process of taking and transmitting images [38,39]. The photography instructions and the process to upload images were iteratively refined and then pre-tested with subsequent participants. Pre-testing sessions were audio-recorded with the participant's consent. Patient demographic, clinical and operative details were collected at the time of recruitment and pre-testing.

### 2.2 Phase 2 – remote testing and evaluation

A prospective cohort study with a new sample of patients who had recently undergone abdominal and vascular surgery was conducted to test the photography instructions and process for transmitting images remotely. Identification and recruitment of patients was identical to the pre-testing phase. Patients could be approached and recruited either before surgery, or after surgery before they were discharged from hospital, with no specified minimum or maximum length of time after surgery for recruitment. Recruitment took place between January and June 2019. Patient demographic, clinical and operative details were collected by a research nurse or surgeon at the participating centre at the time of recruitment. The number of patients approached and recruited were recorded, with reasons for declining the study where applicable. Written informed consent was taken by the research nurse or surgeon at the time of recruitment.

#### 2.2.1 Feasibility, acceptability and usability

Approximately two to three weeks after surgery (or later if the patient had a prolonged stay in hospital), written study information and a paper copy of the photography instructions were posted to the participant's address. The two-to three-week timeframe was chosen as it was relevant to the common recognised timeframe for SSI assessment of within 30 days after surgery, making it relevant for potential implementation in routine practice or SSI surveillance. If participants had a prolonged stay in hospital beyond three weeks they were sent the information once the study team had been notified of their discharge date. A personalised hyperlink to access the online survey was sent by email to coincide with receiving the photography instructions by post. Included in the survey were step-by-step instructions on how to upload and transmit the wound image (or multiple images if patients had more than one wound), items to collect data on patients' familiarity and experience with technology and a set of ‘debriefing’ questions to collect information on how long it took to take the wound images and whether help was required. A box for free-text responses was also provided. Reminder emails or telephone calls were made to those who had not responded within five days.

A sub-sample of participants were selected for a follow-up telephone call to elicit further information on any problems encountered when taking and transmitting images remotely. Included were participants who had indicated a problem in their online survey responses (for example, comments in the free text space), and a convenience sample selected for general feedback.

Feasibility, usability and acceptability were examined. Evaluation metrics included i) participants' response rates to the survey, ii) time taken (days) to respond, iii) the number of participants who were able to successfully a) take and b) transmit an image of their wound images via the online system, iv) efficiency, measured by patient-report of the time taken to take and transmit an image, and whether help was needed. Any problems reported by patients in free text in the online survey, and/or in reminder and follow-up telephone calls, were examined to further evaluate usability and acceptability. This study was conducted entirely separately to patients’ usual care and did not substitute or link with routine follow up and assessments.

#### 2.2.2 Assessment of image quality

Quality of the images received was independently assessed by three clinical reviewers (two general surgeons and one vascular surgeon, qualified for 19, 16 and 15 years respectively), all of whom were experienced in post-surgical follow-up and assessing wounds for SSI. Wound images were viewed on a desktop computer screen, magnifying images using the zoom function if required. The study researcher observed the reviewer as they viewed each image and asked the reviewer to verbally respond to the question “Is the image sufficient to appropriately assess the wound for surgical site infection?” (yes/no). Reasons for responding “no” (i.e., assessing images as inadequate) were further recorded. During analysis, these reasons were subsequently classified by the study researcher with discussion with members of the wider study team (KA and JMB) as either: i) a non-fundamental reason that could be addressed through modification of the method (for example, ensuring the image has better focus or lighting) or; ii) a fundamental reason that could not be addressed/resolved through modification of the method (for example, the wound being obscured by part of the body).

### 2.3 Statistical analysis

Descriptive statistics were used to summarise patient demographics, clinical and operative data, and familiarity and experience with technology. Chi-squared tests were used to compare demographic and operative characteristics for participants who successfully took and transmitted images and those that did not. Inter-rater agreement on the quality of the images was compared using percentage agreement and Krippendorff's alpha coefficient (a recommended measure to use when rating categories are nominal and when no missing data exist) [40]. Values between 0.4 and 0.75 were considered to indicate intermediate to good agreement [40]. Analyses were performed using STATA software version 14.0 [41].

## 3 Results

### 3.1 Phase 1 - development and pre-testing

#### 3.1.1 Literature review to identify key components of wound photography

Data from 11 existing wound photography documents were extracted. Source documents included i) guidelines for medical photographers/healthcare practitioners (n = 1) [29], ii) publications/studies reporting the use of digital images in wound care (n = 7) [30,[42], [43], [44], [45], [46], [47]] and iii) unpublished research study documents (photography protocols/standard operating procedures) where wound images had been used for outcome assessment (n = 3) [[48], [49], [50]]. Data were grouped into 21 categories relevant to taking wound images. Of these, 16 were considered relevant for inclusion in photography instructions for patients using cameras on their own mobile device (Supplementary Table 1).

#### 3.1.2 Pre-testing the photography and processing method

A total of 16 participants pre-tested the wound photography instructions and process for transmitting images. Demographic and clinical details of the study sample are presented (Table 1). The median time since surgery was 28 days (interquartile range 23–44 days). The majority (n = 13, 81.3%) of participants’ wounds had healed. During the iterative process of pre-testing and refinement, four versions of the photography instructions (comprising 43 individual changes) were pre-tested. Most individual changes (n = 22) were to language and wording to improve comprehension. Other changes included streamlining/reducing text and improvements to formatting of the paper document. Final photography instructions for patients (version after pre-testing) are available in Supplementary Fig. 1.

**Table 1:** Patient demographic and clinical details for pre-testing and remote testing study phases.

Demographic/characteristic		Pre-testing sample n = 16		Remote testing sample n = 89	
**Sex, n (%)**	Female	11	(68.8)	40	(44.9)
	Male	5	(31.3)	49	(55.1)
**Age in years, n (%)**					
18 to 35		4	(25.0)	26	(29.2)
36 to 50		4	(25.0)	13	(14.6)
51 to 70		5	(31.3)	33	(37.1)
Over 70		3	(18.8)	17	(19.1)
**Ethnicity, n (%)**					
White/White British		15	(93.8)	87	(97.8)
Asian/Asian British		1	(6.3)	1	(1.1)
Mixed/multiple ethnic groups		0	(0)	1	(1.1)
**Time since surgery in days, n (%)**					
7 to 14		1	(6.3)	3	(3.4)
15 to 30		10	(62.5)	68	(76.4)
more than 30		5	(31.3)	18	(20.2)
**Type of surgery, n (%)**					
General		15	(93.8)	81	(91.0)
Vascular		1	(6.3)	8	(9.0)
**Location of wound(s), n (%)**					
Abdomen		15	(93.8)	80	(89.9)
Leg		1	(6.3)	4	(4.5)
Armpit/Chest		0	(0)	2	(2.3)
Back		0	(0)	1	(1.1)
Groin		0	(0)	1	(1.1)
Neck		0	(0)	1	(1.1)

**Number of wounds, median (IQR)**		3	(3–4)	3	(1–5)

**Urgency of surgery, n (%)**					
Elective		11	(68.8)	58	(65.2)
Unplanned		5	(31.3)	31	(34.8)

**Modality of surgery, n (%)**					
Open		9	(56.3)	47	(52.8)
Laparoscopic		4	(25.0)	39	(43.8)
Laparoscopic converted to open		3	(18.8)	3	(3.4)

**Living status after leaving hospital, n (%)**^a^					
Living with others		Not collected		76	(86.4)
Living alone		Not collected		12	(13.6)

a Data available for 88/89 in remote testing sample.

### 3.2 Remote testing & evaluation

#### 3.2.1 Participants

Some 129 patients were approached to test the method remotely of which 116 (90.0%) were eligible. Of these, 91/116 (78.4%) consented to participate and 89 (76.7%) were included in the analysis. Reasons for exclusion and declining participation are reported (Supplementary Fig. 2). Demographic and clinical details of included participants are presented (Table 1). Median time between patients’ surgery and the invitation to take and transmit images was 22 days (interquartile range 20–27 days).

#### 3.2.2 Feasibility

**Fig. 1:**
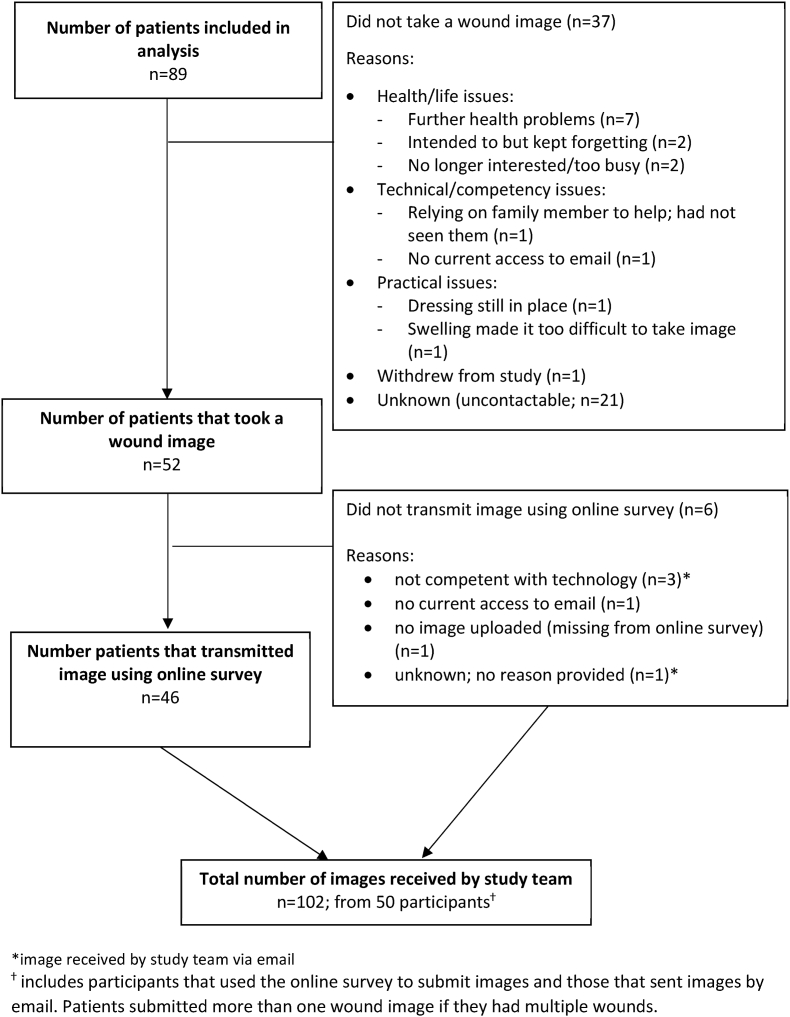
Flow diagram of participants taking and transmitting images remotely *image received by study team via email ^ⴕ^ includes participants that used the online survey to submit images and those that sent images by email. Patients submitted more than one wound image if they had multiple wounds.

Fifty-two of 89 (58.4%) participants took one or more wound image(s) and 46/52 (88.4%) successfully transmitted the image(s) (Fig. 1). No differences were observed in demographic or clinical characteristics for participants who took and transmitted an image and those who did not (Supplementary Table 2). Median time to receive an image from participants was four days (inter-quartile range one to 10 days). Fifty-six (62.9%) participants required a reminder to send images due to no response at seven days. Reminders were effective (that is, resulted in an image being transmitted) for 19/56 (33.9%) participants. Where it was possible to ascertain reasons for not taking or transmitting an image through telephone follow-up or in reply to reminders (n = 16/37, 43.2%), reasons were primarily further health problems or lack of time/low priority of the study compared to other demands (n = 11/16; 68.8%). Only a small minority (n = 4/16; 25.0%) of patients reported practical or technical problems that made it impossible to take or transmit an image at all (Fig. 1). Specific details of problems encountered are described below.

#### 3.2.3 Usability and acceptability

Responses to debriefing questions as part of the online survey demonstrated that, for those who were able to take and transmit an image of the wound, the process was quick to do. Most (n = 41; 87.2%) reported that it took less than five minutes to take a clear wound image. Level of experience with using a mobile device was generally high or moderate in those who successfully transmitted images (Supplementary Table 3). Most participants reported that they were experienced with using their device to take photographs and did so on a daily or weekly basis (n = 12; 26.1% and n = 24, 52.2%, respectively). Nineteen participants (40.4%) reported that they took the wound image(s) themselves and 25 (53.2%) reported that someone else took the image(s) (Supplementary Table 3). The remaining three (6.4%) participants reported taking some images themselves while someone else took others. These participants had more than one wound.

Only 15 problems from 13 different participants were reported in total, either via the online survey or during reminder/follow-up telephone calls (Supplementary Table 4). Many of these problems, however, were overcome and only four participants were not able to take or transmit an image at all, as described above. Six participants (6.7%) were able to take an image(s) but did not transmit them using the online survey. Reasons related to technical/technical competency difficulties or not attempting the online system. Images were instead sent to the study team by email (self-initiated by the participants and not suggested by the study team). Three participants described problems with taking images when the wound was in a difficult location, although for two of the three participants these problems were overcome with help from others to take the image. A minority of participants (n = 8; 17.0%) reported in the online survey that they needed help to transmit the image. In all cases they were all able to ask a family member to assist. Positive feedback was reported without prompting in the online survey from nine patients. Examples included describing the process as “*easy*”, “*straightforward*” and that “*instructions were clear*”.

#### 3.2.4 Image quality

A total of 102 images were received from 50 participants. Quality of the images was overall very high. Ninety-eight (96.1%) images were judged to be sufficient for potentially assessing the wound for SSI by at least one of the three reviewers and 87 (85.3%) images were judged to be sufficient by at least two of the three reviewers. Seventy-five (73.5%) images were unanimously considered to be sufficient by all three reviewers. Only four (3.9%) images (from four different patients) were considered inadequate by all three reviewers. Statistical analysis of agreement between reviewers' judgements of image quality demonstrated 85% overall agreement with a Krippendorff's alpha co-efficient of 0.41, indicating intermediate to good agreement.

Most reasons for judging images as inadequate were classified by the study team as non-fundamental and likely could be overcome in further attempts to photograph the wound. Examples included poor focus, poor camera positioning/angle or presence of a shadow over the wound. A minority of images (n = 8), however, were considered inadequate for potentially assessing the wound for SSI by at least one reviewer for a more fundamental reason that could not be overcome by the photography method. These were all wounds on a difficult site on the body to image and part of the wound was hidden from view, for example, within the umbilicus (belly button).

## 4 Discussion

This study has developed and evaluated a feasible and acceptable method for obtaining clear, standardised digital images of wounds from patients using their own mobile devices for remote SSI assessment after leaving hospital. Clear, comprehensive instructions that are acceptable to patients have been developed and tested and were demonstrably effective in producing high quality images that are fit for purpose to potentially assess the wound for SSI. Few technical or competency problems were reported by participants, serving as proof-of-concept for the feasibility and acceptability of using patient-generated digital wound images for remote SSI assessment. The photography instructions and process for patients to transmit images are reproducible and now ready for use in research studies and routine follow-up.

Few other studies have reported on the quality of patient-taken images for potential SSI assessment, and typically these have been studies that focused on acceptability and/or usability of an app [12,13]. These studies provided training for patients to use the app and an opportunity to practice taking and uploading images during their inpatient stay. Our results are comparable to that reported by de Heide et al. [13], which provided prior training and image-taking practice for patients and demonstrated that 88% of images of cardiology wounds were of sufficient quality for assessment. Our study has demonstrated that patient-generated, standardised images of adequate quality can be obtained using our written instructions without the need for prior training, therefore providing good data with fewer resources required. Where other studies including patient-taken images reported data on response rates, this typically reflected the overall response rate for use of an app or online tool (i.e. for patients to remotely submit data more broadly and not specific to taking and transmitting a wound image). Data was often lacking on the number of responses that specifically included an image upload. Furthermore, some studies included multiple timepoints, for example, requiring participants to take daily photographs of their wound for 30 days [23,25] rather than a single timepoint as in the current study. Response rates are, therefore, variable in other studies. Findings range from 58/223 (26.0%) full adherence and 93/223 (41.7%) partial adherence to a smartphone wound assessment tool [23], 51/70 (73%) for patients completing an online follow-up visit [11] and 58% and 82% for orthopaedic and breast patients, respectively, in feasibility study of a mobile app for post-operative monitoring of patients [25]. Direct comparison with the response rate in the current study is less informative due to these different study designs. The current study, however, adds to existing literature by reporting on response rates for uploading wound image(s) for a single timeframe and image quality without using an app, and is therefore useful for application to routine clinical practice. The method used in the current study and the standardised photography instructions are reproducible with generic application for use with any mobile device that has an in-built camera and ability to connect to the internet, without the need to download or maintain additional software.

In the UK, the National Wound Care Strategy Programme (NWCSP) commissioned by NHS England and NHS Improvement has recently published recommendations and practical guidance for health and care professionals for the use of digital images in wound care [31]. The recommendations seek to promote and standardise the practice of taking good quality, clear images and include step-by-step instructions for healthcare workers to take digital wound images. This supports the increasing awareness that digital images are moving towards being part of standard care with increasing recognition of the benefits for healthcare providers and patients. The wound photography instructions in the current study further add to this movement by developing and testing instructions specifically for use by patients. The instructions were primarily developed for remote assessment of SSI; however, they are applicable to other areas of wound care and research and supplement the work of the NWCSP and use of digital images in practice going forward.

This study used robust methodology to develop photography instructions for patients that were informed by the literature, pre-tested and finally testing remotely with a large sample of participants. A range of patients undergoing a range of different planned and non-planned procedures were included. Wounds varied in size and location on the abdomen, and the included patients were diverse in their age, gender and experience in using smartphones and taking images. This supports the validation and generalisability of the work, increasing its relevance to the wider population. There are, however, some limitations. Most patients were white/white British, and the acceptability and feasibility of the method in other ethnic or cultural groups is unclear. The method was developed and tested with patients undergoing a range of abdominal and vascular surgeries for practical, clinical, and logistical reasons. While the applicability of the method to other surgical specialties is anticipated, further work is needed to evaluate the feasibility of collecting wound images from a wider group of patients undergoing a wider range of surgical procedures and with different wound types and locations. Findings are currently limited to evaluation of this method at a single timepoint two-to-three weeks after surgery, and the feasibility of the method for obtaining wounds images sooner after patients leave hospital warrants further investigation. Further work is also warranted to explore the application of the method in areas of wound care which may require longer timeframes than the 30-day period typical for SSI assessment, for example, remote assessment for wound healing/monitoring. Some 37/89 (41.6%) participants in the study did not take or transmit a wound image. When it was possible to ascertain reasons, few technical or practical problems were reported. However, for almost a quarter of the participants (n = 21; 23.6%), no images were received and efforts to reach participants by telephone or email were unsuccessful. Reasons for non-compliance and/or whether attempts to take an image had been made by these participants and were unsuccessful are, therefore, unknown. Without this data, conclusions that can be drawn from the findings are limited. Further uncertainty on the generalisability and acceptability of the method arises from patients that declined the invitation to take part in the study. Patients may have declined to participate because, for example, they found the experience of seeing their wound upsetting and were therefore not willing to photograph it. Despite the growing and encouraging evidence to support remote wound assessment and the inclusion of images, it is important to acknowledge that not all wounds are suitable for remote assessment using patient-taken images as demonstrated in the current study. To adequately view and assess the entire wound may in some cases require manipulation of the skin which may be more appropriate to be performed by clinical staff rather than by a patient/carer themselves to the avoid risk of disturbing the wound or introducing infection.

Other recent advances in methods to assess SSI remotely include a newly developed and validated SSI outcome measure, suitable for patient and/or healthcare professional completion [[51], [52], [53]]. With an effective system in place to collect and analyse data from such an outcome measure in real-time, such as automated notification of scores over a pre-defined threshold, remote assessment has the potential to identify problems sooner before they extenuate. This may lead to faster treatment compared to the delay that may occur when scheduling in-person appointments. Recommendations for future work include a full evaluation of remote wound assessment as the next step following this initial feasibility and acceptability study. The method could be used and evaluated in practice, for example, as a method for outcome assessment in a clinical trial and/or to assess SSI in clinical practice. Work is needed to explore, for example, whether images in combination with data collected by the outcome measure has added value for remote SSI assessment. Being able to review images alongside other patient-reported information on symptoms may, for example increase accuracy of SSI diagnosis when the score on the outcome measure is borderline of the SSI cut-off threshold. Exploring the utility of remote assessment with patients with different demographics, including different ethnic and cultural groups, different types of wounds and undergoing other types of surgery, using qualitative methods for example, is required to determine how useful the methods are in patient populations other than that included in the current study. In particular, it is important to understand the challenges experienced by those who did not participate and how this compares to attendance for a face-to-face follow-up assessment. Findings will inform the design principles for future initiatives and methods for remote assessment of wounds after hospital discharge in trials, routine follow-up and SSI surveillance. Attention should be paid to exploring how to optimise access to required digital technologies, to maximise equality and inclusion for patients.

## Source of funding

The Selfi-wound study: self-taken images of surgical wounds was supported by the National Institute for Health and Care Research (NIHR) Bristol Biomedical Research Centre (BRC) at the University Hospitals Bristol and Weston NHS Foundation Trust and the University of Bristol, the Royal College of Surgeons of England Bristol Surgical Trials Centre and the Medical Research Council (MRC) ConDuCT-II (Collaboration and innovation for Difficult and Complex randomised controlled Trials In Invasive procedures) Hub for Trials Methodology Research (MR/K025643/1). The views and opinions expressed in this publication are those of the authors and not necessarily those of the UK National Health Service, the National Institute for Health Research, the Department of Health and Social Care, the Royal College of Surgeons of England or the MRC.

## Declaration of competing interest

The authors have no conflicts of interest to declare.
