# Supplementary material for: Remote assessment of surgical site infection (SSI) using patient-taken wound images: Development and evaluation of a method for research and routine practice
Source: J Tissue Viability. 2023 Feb;32(1):94–101. doi: 10.1016/j.jtv.2023.01.001 (PMC10322728; doi:10.1016/j.jtv.2023.01.001)
Supplement: Multimedia component 1 [file mmc1.docx]

**Supplementary files**

Supplementary table 1. Identified components key for taking wound images

|  | **Key component** | **Source** | | | **Verbatim example** | **Component included in preliminary version of photography instructions for patients** |
| --- | --- | --- | --- | --- | --- | --- |
|  |  | **Professional guidelines (n=1)** | **Published paper/study* (n=7)** | **Unpublished research protocol* (n=3)** |  |  |
| 1 | Protecting dignity | ✓ | ✓ | ✓ | “Extraneous clothing should be removed by the patient (or carers) but the patients’ dignity should not be compromised.” | ✓ |
| 2 | Preparation: wound cleaning | ✓ | ✓ | ✓ | "In general, wounds and the surrounding area (particularly the perineum) should be cleaned before photography; otherwise there may be confusion as to the condition and extent of the wound." | ✓ |
| 3 | Preparation: dressing removal | x | ✓ | ✓ | “If the patient has a dressing in situ, photographing the wound should coincide with planned dressing changes” | ✓ |
| 4 | Setting: where the image is taken | ✓ | ✓ | x | “Wherever possible, take the photo in a treatment room because the overhead lighting produces a better picture.” | ✓ |
| 5 | Equipment: type of camera | ✓ | ✓ | ✓ | "Consideration must be made about whether photographs are taken on cameras or phones and whether the device used is owned by the clinician or the Trust" | ✓ |
| 6 | Lighting/Flash | ✓ | ✓ | ✓ | “A flash is used in all settings to ensure adequate and consistent lighting” | ✓ |
| 7 | Background | ✓ | ✓ | ✓ | “Backgrounds should be plain and unobtrusive providing no distraction from the area of interest” | ✓ |
| 8 | Position of participant | ✓ | ✓ | ✓ | “comfortably positioned in the correct anatomical position” | ✓ |
| 9 | Scaling tool (i.e. ruler) | ✓ | ✓ | ✓ | “A scaling tool is advised as appropriate” | ✓ |
| 10 | How images are transmitted | ✓ | ✓ | ✓ | “Photos should be immediately uploaded, deleted from the camera and/or device and shared drive once this is completed” | x |
| 11 | Storage | ✓ | ✓ | ✓ | “stored securely and disposed of securely when no longer required” | x |
| 12 | Patient identifier | x | ✓ | ✓ | “Before photos were taken, a 15-cm ruler with clear millimeter divisions was placed next to the wound as well as a patient identification number and the date of the assessment” | x |
| 13 | Distance from wound/framing | x | ✓ | ✓ | “The photograph is framed by altering the distance between the lens and the wound or using the zoom function if present” | ✓ |
| 14 | Angle/plane of camera to wound | ✓ | ✓ | ✓ | “The camera should be held perpendicular to the wound” | ✓ |
| 15 | Focus | x | ✓ | ✓ | “Auto focus” | ✓ |
| 16 | Reviewing the image | x | ✓ | ✓ | “Review the picture on the back of the camera” | ✓ |
| 17 | Multiple images | x | ✓ | ✓ | “A second photograph of all wounds is obtained to ensure at least 1 good-quality photograph” | ✓ |
| 18 | Shadow | x | ✓ | x | “There should be no areas of shadow” | ✓ |
| 19 | Resolution | x | ✓ | x | “Good resolution (usable file size) and quality depends on the equipment used.” | x |
| 20 | Colour control/calibration | ✓ | ✓ | ✓ | “Where colour is an important factor, it is useful (where practicable) to include a calibrated colour chart and/or grey card in the frame or at the beginning of a series of images.” | x |
| 21 | Use of mirror as a prop | ✓ | x | x | “If a wound is located in an awkward position it may be helpful to use a large dental mirror” | ✓ |

*component mentioned in at least one publication

Supplementary figure 1: Photography instructions for patients (final version after pre-testing)


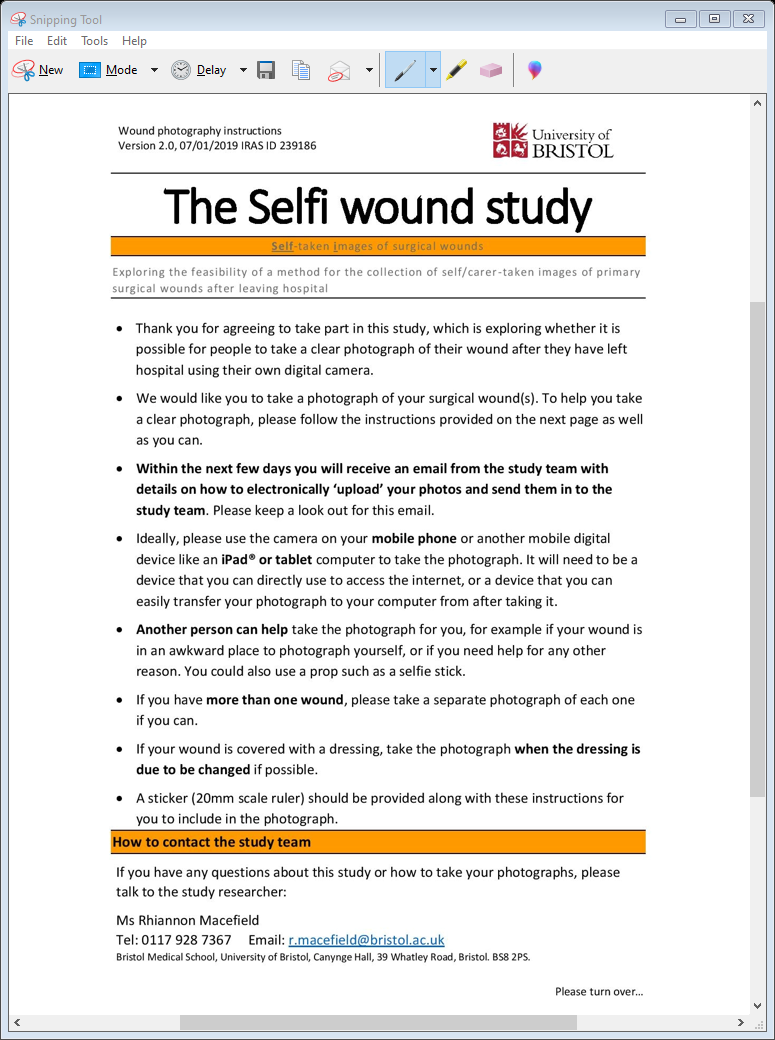


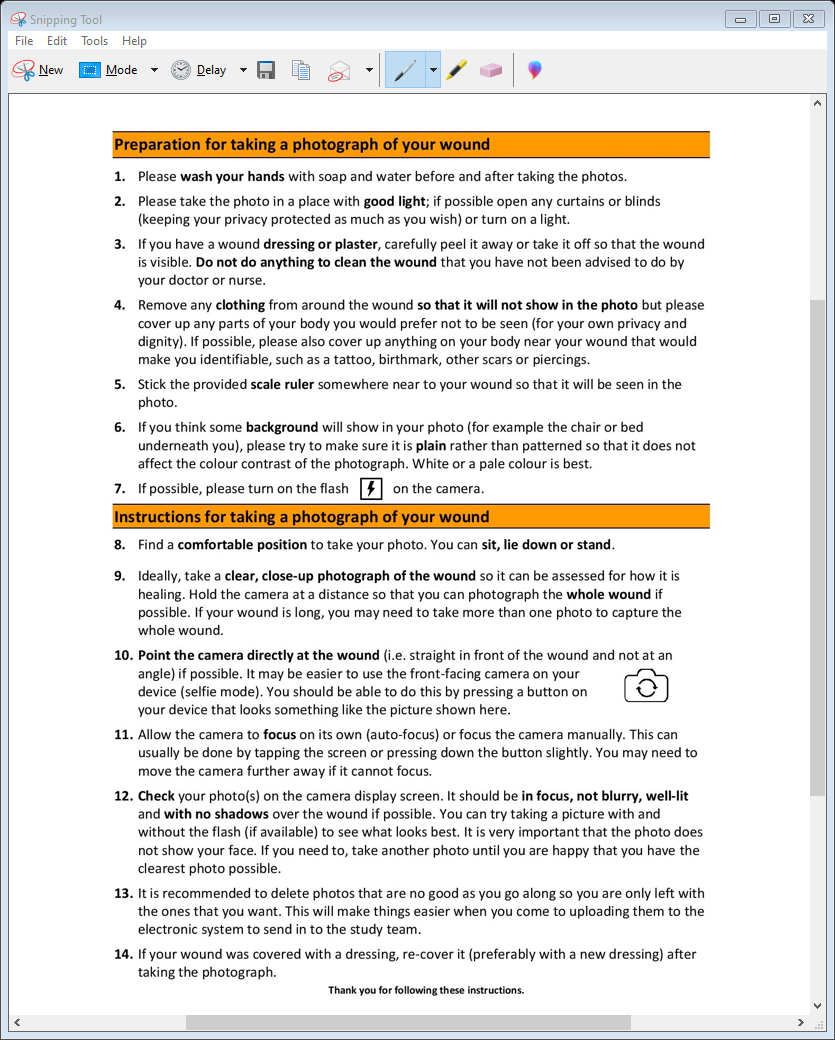


Supplementary figure 2. Recruitment for the remote-testing phase of the study

Eligible (n=116)

Excluded (n=2)

- Did not provide contact details (n=1)
- Extended hospital stay (n=1)

Consented (n=91)

Ineligible/excluded (n=13)

- No access to camera/internet (n=10)
- Administration error – missed (n=3)

Approached and invited to participate (n=129)

Included in analysis (n=89)

Declined (n=25)

- Too much on (n=6)
- Not willing to photograph wound (n=2)
- Not interested in participating (n=2)
- Further health issues (n=1)
- Dislike of using online systems (n=1)
- Did not want to be followed up (n=1)
- Not competent with technology (n=1)
- Consent form issues (n=1)
- Reason not recorded (n=10)

Supplementary table 2. Comparison of demographic and clinical details for patients who did and did not take and transmit a wound image.

| **Demographic/characteristic** | **Patients who took and transmitted a wound image**  **n=46** | **Patients who did not take and transmit a wound image**  **n=37** |
| --- | --- | --- |
| **Sex, n (%)** |  |  |
| Female | 19 (41.3) | 18 (48.7) |
| Male | 27 (58.7) | 19 (51.4) |
| **Age in years, n (%)** |  |  |
| 18 to 35 | 13 (28.3) | 13 (35.1) |
| 36 to 50 | 7 (15.2) | 6 (16.2) |
| 51 to 70 | 19 (41.3) | 11 (29.7) |
| Over 70 | 7 (15.2) | 7 (18.9) |
| **Ethnicity, n (%)** |  |  |
| White/White British | 45 (97.8) | 37 (100.0) |
| Asian/Asian British | 0 | 0 |
| Mixed/multiple ethnic groups | 1 (2.2) | 0 |
| **Time since surgery in days, n (%)** |  |  |
| 7 to 14 | 2 (4.3) | 1 (2.7) |
| 15 to 30 | 34 (73.9) | 29 (78.4) |
| more than 30 | 10 (21.7) | 7 (18.9) |
| **Type of surgery, n (%)** |  |  |
| General | 43 (93.5) | 34 (91.9) |
| Vascular | 3 (6.5) | 3 (8.1) |
| **Location of wound(s), n (%)** |  |  |
| Abdomen | 42 (91.3) | 33 (89.2) |
| Leg | 3 (6.5) | 0 |
| Armpit/Chest | 0 | 2 (5.4) |
| Back | 1 (2.2) | 0 |
| Groin | 0 | 1 (2.7) |
| Neck | 0 | 1 (2.7) |
| **Number of wounds, median (IQR)** | 3 (1 to 4) | 3 (1 to 6) |
| **Urgency of surgery, n (%)** |  |  |
| Elective | 29 (63.0) | 25 (67.6) |
| Unplanned | 17 (37.0) | 12 (32.4) |
| **Modality of surgery, n (%)** |  |  |
| Open | 25 (54.3) | 18 (48.7) |
| Laparoscopic | 19 (41.3) | 18 (48.7) |
| Laparoscopic converted to open | 2 (4.3) | 1 (2.7) |
| **Living status after leaving hospital, n (%)** |  |  |
| Living with others | 39 (84.8) | 32 (86.5) |
| Living alone | 7 (15.2) | 4 (10.8) |
| missing | 0 | 1 (2.7) |

Supplementary table 3. Patients’ experience and frequency of using mobile devices and taking images.

|  | **Pre-testing sample n=16** | **Remote testing sample**^ⴕ^ **n=47** |
| --- | --- | --- |
| **Level of experience using mobile device, n (%)*** |  |  |
| Very experienced/expert | 6 (37.5) | 21 (44.7) |
| Somewhat/moderately experience | 8 (50.0) | 24 (51.1) |
| Not experienced | 1 (6.3) | 2 (4.3) |
| **Frequency using phone/tablet to take photographs, n (%)**** |  |  |
| Everyday | 5 (31.3) | 12 (26.1) |
| Weekly | 4 (25.0) | 24 (52.2) |
| Monthly | 5 (31.3) | 8 (17.4) |
| Yearly | 0 | 1 (2.2) |
| Never | 0 | 1 (2.2) |
| **Time taken to take the wound photograph, minutes** |  |  |
| Less than 5 | n/a | 41 (87.2) |
| 5 to 10 | n/a | 5 (10.6) |
| 10 to 15 | n/a | 0 |
| More than 15 | n/a | 1 (2.1) |
| **Photographer** |  |  |
| Patient (self-taken) | n/a | 19 (40.4) |
| Other (family member/friend) | n/a | 25 (53.2) |
| Patient and other | n/a | 3 (6.4) |
| **Help needed to transmit the image** |  |  |
| No | n/a | 39 (83.0) |
| Yes | n/a | 8 (17.0) |

^ⴕ^ patients who completed the online survey n=47

* data available for 15/16 in pre-testing sample

** data available for 14/16 in pre-testing sample and 46/47 in remote testing sample

“n/a” indicates not assessed in pre-testing study

Supplementary table 4. Summary of patient-reported issues/barriers to taking and transmitting wound images

| Reported issue/barrier (number reporting problem) | Consequence of issue/barrier |
| --- | --- |
| - Difficult location of wound/swelling (n=1) | Image capture prevented (n=4) |
| - Dressing in place for prolonged length of time (n=1) |  |
| - Relying on family member to help and not able to see them (n=1) |  |
| - No access to email (n=1) |  |
| - Problems finding the image on device (n=1)* - Error (no image uploaded but patient thought it had) (n=1) - No current access to email (n=1) - Inexperienced with technology (n=1)* - Link for survey not noticed in invitation email (n=1)* - No attempt to use online system (no reason provided)* (n=1) | Image captured but transmission prevented (n=6) |
| - Location of wound difficult to take image without help (n=2) - Problem with the online system crashing (n=1) - Dressing in place (n=1) - Link did not access online system on first attempt (n=1) | No consequence; issue/barrier reported but did not prevent image capture or transmission (n=5) |

*Image sent to study team by email
